# Anesthetic Management of High-Output Heart Failure in Aortocaval Fistula Using Intra-aortic Balloon Occlusion: A Case Report

**DOI:** 10.7759/cureus.88644

**Published:** 2025-07-24

**Authors:** Keisuke Sumii, Hiroe Hamaguchi, Koichiro Goto

**Affiliations:** 1 Anesthesiology, Saitama Sekishinkai Hospital, Sayama, JPN

**Keywords:** aortocaval fistula, congestive heart failure (chf), intra-aortic balloon occlusion, ruptured abdominal aortic aneurysm, shunt blood flow

## Abstract

An aortocaval fistula caused by rupture of a right common iliac arterial aneurysm is a rare but potentially fatal condition that can progress to acute heart failure and even multi-organ failure due to abnormal shunting of blood through the fistula. We report a case of a patient with such a fistula who was rapidly diagnosed, and following emergent surgical consultation, underwent intra-aortic balloon occlusion prior to laparotomy. This intervention promptly stabilized the patient’s circulation by addressing the high-output heart failure.

## Introduction

Aortocaval fistula (ACF), an abnormal communication between the aorta and the inferior vena cava (IVC), has several causes, including abdominal trauma, iatrogenic injury from lumbar disc surgery, and tumor invasion near the abdominal aorta and IVC. The most common cause of ACF is rupture of an abdominal aortic aneurysm. Previous reports estimate that the incidence of ACF due to spontaneous rupture of an abdominal aortic aneurysm ranges from 0.2% to 6%, with a mortality rate of approximately 30% [1,2].

For diagnosis, we use contrast-enhanced CT, MRI, and ultrasonography. Contrast-enhanced CT is typically the first diagnostic modality, as it allows comprehensive assessment of the ACF’s structure. Additionally, 3D imaging offers high spatial resolution and helps delineate the anatomy essential for surgical or endovascular planning. However, a limitation of contrast-enhanced CT is that it cannot be used in patients with contrast media allergies [3].

Contrast-enhanced MRI has been reported to quantify fistula flow [4]. MRI also offers superior soft tissue contrast and has been found useful in detecting graft infections, which are challenging to identify with contrast-enhanced CT [5]. A disadvantage of MRI, however, is the longer imaging time required. Ultrasonography is a rapid diagnostic tool that can be used in patients with contrast allergies and enables direct visualization of fistula flow [6]. However, its effectiveness may be limited in cases where intestinal gas or retained tissue obscures the deep abdomen [5,7]. Of the three modalities mentioned, only contrast-enhanced CT was used in this case. The key clinical finding was the simultaneous enhancement of the right common iliac artery and IVC during the arterial phase [8-10].

Among the complications associated with ACF, high-output heart failure due to increased venous perfusion can progress rapidly and is considered a poor prognostic factor [11,12]. In this case, the fistula originated not from an aortic aneurysm but from a common iliac artery aneurysm. Although the connection between the common iliac artery and either the common iliac vein or IVC is rare, a few cases have been reported [13].

There are two primary treatment options for ACF: open surgical repair and endovascular repair. Endovascular treatment has recently gained attention due to its shorter operative time, reduced blood loss, and less postoperative pain. However, in this case, the aneurysm was located in an anatomically challenging region involving the common iliac artery. Therefore, open surgery - an approach familiar to the treating institution - was selected.

Intra-aortic balloon occlusion (IABO) is a technique used to control life-threatening bleeding by inflating a balloon catheter within the aorta. By using IABO to temporarily occlude the fistula and promptly address the high-output heart failure, we were able to stabilize the patient’s circulation and ultimately save their life.

## Case presentation

**Figure 1:**
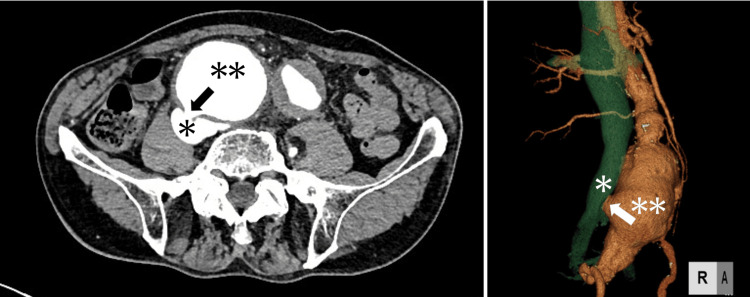
Left: Arterial phase of abdominal contrast-enhanced CT at presentation (horizontal section at the L5 level). Right: 3D-CT reconstruction In the left image, a fistula is visible between the right common iliac artery and the IVC, as the IVC is enhanced during the arterial phase. A single asterisk marks the IVC, while a double asterisk indicates the right common iliac artery (either black or white, depending on the image contrast). The large fistula, caused by rupture of a right common iliac arterial aneurysm (black arrow), led to increased venous perfusion, resulting in high-output heart failure. In the right image, a 3D-CT reconstruction shows the right common iliac artery (red) and IVC (green). The right common iliac artery aneurysm is in contact with the IVC, forming an ACF (white arrow). ACF, arteriovenous fistula; IVC, inferior vena cava

A male patient in his 70s (height: 167 cm; body weight: 57 kg) with no known medical history was transported to our hospital by emergency ambulance due to respiratory distress and nausea that had developed earlier that day. On arrival, his vital signs were recorded: he was conscious, with a heart rate of 113 beats per minute (sinus rhythm), a blood pressure of 98/44 mmHg, an SpO₂ of 97% on room air, and a respiratory rate of 30 breaths per minute. Electrocardiography revealed a complete right bundle branch block. A chest X-ray showed a cardiothoracic ratio of 55% and bilateral pulmonary congestion. Contrast-enhanced CT revealed a ruptured right common iliac arterial aneurysm with a 7.5 mm fistula between the right common iliac artery and the IVC at the L5 level, prompting emergency abdominal surgery (Figure 1).

An IABO was inserted via the right femoral artery under local anesthesia with 1% lidocaine, and the fistula was occluded from the arterial side. Immediately after fistula occlusion, systolic blood pressure increased from 65 mmHg to 126 mmHg, and central venous pressure (CVP) decreased from 19 mmHg to 13 mmHg. Regional cerebral oxygen saturation (rSO₂) increased from 44% to 65% on the right and from 37% to 64% on the left (Table 1).

**Table 1:** Changes in SBP, CVP, and rSO₂ (right and left) before and after fistula plugging using IABO Immediately after fistula occlusion, SBP increased from 65 mmHg to 126 mmHg, while CVP decreased from 19 mmHg to 13 mmHg. rSO₂ in the right and left hemispheres rose from 44% to 65% and from 37% to 64%, respectively. CVP, central venous pressure; IABO, intra-aortic balloon occlusion; rSO₂, regional cerebral oxygen saturation; SBP, systolic blood pressure

Parameter	Before fistula plugging	After fistula plugging
SBP (mmHg)	65	126
CVP (mmHg)	19	13
rSO₂ (right, %)	44	65
rSO₂ (left, %)	37	64

After hemodynamic stabilization, general anesthesia was induced with 70 mg of rocuronium, followed by 200 mg of propofol and 0.1 mg of fentanyl. Anesthesia was maintained with 1-1.5% sevoflurane and continuous infusion of remifentanil at 0.12-0.15 mcg/kg/min. The infrarenal abdominal aorta at the L2-L3 level was clamped, followed by clamping of the external iliac artery and ligation of the internal iliac artery. The aortic wall was incised, the ACF was ligated and closed, and a Y-graft was placed. During fistula repair, venous bleeding was noted between the IABO and the fistula.

The lactate level measured just before fistula closure was 5.34 mmol/L and gradually decreased to 3.18 mmol/L by the end of the procedure. The operation lasted five hours and 12 minutes, with an estimated blood loss of 5,680 mL. A total of 5,300 mL of extracellular fluid was infused, along with 2,620 mL of autologous blood recovered via a cell saver system, 10 units of fresh frozen plasma, and 20 units of platelet concentrate.

**Figure 2:**
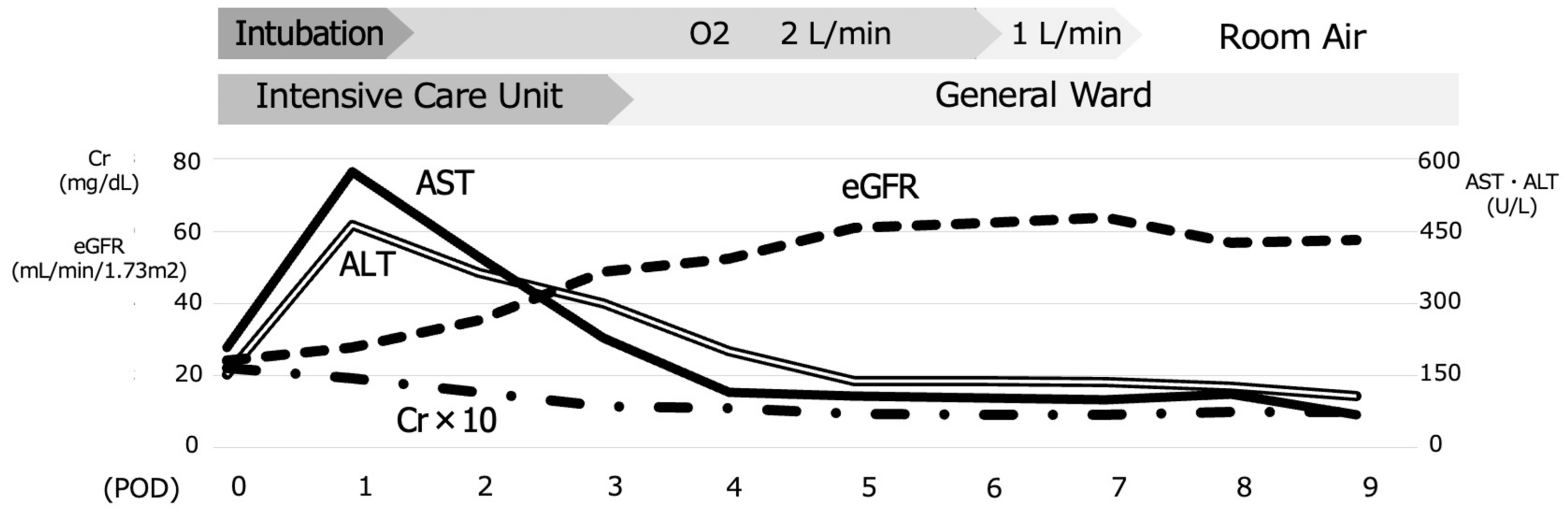
Postoperative course On postoperative day 1, AST (black solid line) and ALT (black double line) peaked and then gradually decreased to within the normal range. Cr (multiplied by 10; black uneven dotted line) and eGFR (black dotted line) were initially at abnormal levels immediately after surgery but showed improvement over the next two to three days. As the patient’s circulation remained stable, he was extubated on postoperative day 2 and transferred to the general ward on postoperative day 3. His overall condition continued to improve without progression to multiple organ failure. ALT, alanine aminotransferase; AST, aspartate aminotransferase; Cr, creatinine; eGFR, estimated glomerular filtration rate

Postoperatively in the ICU, aspartate aminotransferase (AST) and alanine aminotransferase (ALT) peaked on postoperative day 2, while creatinine (Cr) and estimated glomerular filtration rate showed a trend toward improvement. As the patient’s circulation remained stable, he was extubated on postoperative day 2, transferred to the general ward on postoperative day 3, and discharged on postoperative day 22 without neurological sequelae (Figure 2).

A panel of blood tests - including cardiac troponin I, creatine kinase MB, brain natriuretic peptide, AST, ALT, Cr, and eGFR - was performed upon ICU discharge and is compared to values at hospital arrival in Table 2.

**Table 2:** Blood test results at hospital admission, in the ICU (POD1), and in the general ward (POD9) Cardiac troponin I and brain natriuretic peptide were elevated upon arrival, indicating cardiac stress. AST and ALT were also elevated due to hepatic congestion. Cr and eGFR reflected impaired renal function. AST and ALT peaked on POD2 and gradually declined thereafter. Cr and eGFR also showed improvement after POD2. By POD9, most blood test values had returned to near-normal levels. ALT, alanine aminotransferase; AST, aspartate aminotransferase; Cr, creatinine; eGFR, estimated glomerular filtration rate; POD1, postoperative day 1; POD2, postoperative day 2; POD9, postoperative day 9

Test	Reference range	Arrival (hospital)	ICU (POD1)	General ward (POD9)
AST	13-30 U/L	173	478	57
ALT	10-42 U/L	126	385	90
Cr	0.6-1.1 mg/dL (male)	2.2	1.9	1
eGFR	≥60 mL/min/1.73 m²	24	28	58

At six-month follow-up, contrast-enhanced CT showed no endoleak and no abnormalities in the anastomotic region of the artificial graft.

## Discussion

This is an unusual case of a ruptured right common iliac artery-caval fistula that progressed to acute heart failure due to shunt blood flow through a large fistula. Early preoperative diagnosis allowed for fistula occlusion via IABO prior to open surgery, which helped stabilize intraoperative hemodynamics and minimize blood loss.

Rupture of a right common iliac artery aneurysm forming an ACF can lead to congestive heart failure due to the abrupt increase in venous return caused by direct arterial-to-venous shunting [14-16]. This risk is particularly high in patients with large fistulas and significant shunt volumes [17,18]. In this case, although the patient had no history of heart failure or coronary artery disease, preoperative findings - including respiratory distress, elevated BNP, AST, and ALT levels, pulmonary congestion on chest X-ray, and abnormally high CVP - were consistent with a diagnosis of congestive heart failure [19].

Right ventricular enlargement in the context of congestive heart failure can impair left ventricular function through interventricular septal shift [20,21]. Additionally, elevated right atrial pressure reduces coronary sinus perfusion by diminishing the pressure gradient between the coronary arteries and coronary sinus [22]. The inflow of arterial blood into the IVC through the fistula causes both systolic and diastolic pressures to fall, thereby reducing coronary perfusion and contributing further to heart failure [23]. As heart failure progresses, decreased organ perfusion can ultimately lead to multi-organ failure [14].

Unfortunately, no imaging prior to the presented CT scans was available, so we could not evaluate the aneurysm’s progression. However, in the left panel of Figure 1, the right common iliac arterial wall appears to have perforated into the IVC, and the matching density of contrast enhancement between the artery and vein in the arterial phase strongly suggests that the ACF resulted from rupture of the right common iliac artery aneurysm.

The 30-day mortality rate for ACF associated with ruptured aortic aneurysms is as high as 36%, with even higher mortality in cases where the aneurysm is not diagnosed preoperatively [1,24]. Therefore, rapid diagnosis using contrast-enhanced CT and early therapeutic intervention are crucial for survival [25].

Anesthetic management during IABO placement should follow principles similar to those used in managing congestive heart failure. These include (1) avoiding excessive preload; (2) preventing sudden increases in afterload; and (3) maintaining adequate perfusion pressure for coronary and systemic circulation [26,27]. In this case, we administered intravenous fluids carefully, keeping stroke volume variation below 10-15% and avoiding CVP overload. Noradrenaline was administered at approximately 0.15 mcg/kg/min to maintain a target mean blood pressure of 65 mmHg, with an arterial line placed in the emergency room upon the patient’s arrival. After fistula occlusion, lactate levels peaked and rSO₂ improved [28]. Once hemodynamic stability was achieved, we administered furosemide and human atrial natriuretic peptide to reduce cardiac overload. Lactate levels are a marker of improved tissue perfusion, while increased rSO₂ indicates improved cerebral oxygenation [29]. In this case, rSO₂ improved despite no significant change in hemoglobin levels from bleeding or transfusion, and together with improved respiratory status and lactate reduction, this suggests enhanced blood flow and oxygen delivery [30].

IABO is commonly used in trauma cases for proximal vascular control via descending aortic clamping during hemorrhagic shock. Previous reports suggest that reliable hemostasis using IABO can reduce mortality and transfusion needs in such cases [31]. While this case differs from trauma-related hemorrhage, further research is needed to determine the utility of IABO in similar nontraumatic ACFs.

Transesophageal echocardiography can be useful for intraoperative cardiac function assessment and helps guide anesthetic management in heart failure. Although coagulation studies were not performed and there was no CT evidence of esophageal or gastric varices, we did not insert IABO before general anesthesia due to the added risk of gastrointestinal bleeding from possible esophageal varices or portal hypertension associated with right heart failure and anticoagulation (heparin) use [32-35].

There are two primary treatments for ACF: open surgery and endovascular repair. While open surgery was the gold standard until 1998, endovascular repair has recently gained popularity due to shorter operative times, reduced blood loss, and decreased postoperative pain [36,37]. However, despite its widespread adoption for aortic aneurysms, open repair remains essential in many situations [38]. A large cohort study found that although endovascular aneurysm repair (EVAR) results in lower 30-day mortality and perioperative complications, long-term follow-up revealed higher rates of aneurysm rupture and need for reintervention compared to open repair [38]. Therefore, patients undergoing EVAR require long-term imaging follow-up with CT or ultrasound to monitor for complications such as endoleak [39]. In the absence of standardized guidelines, comprehensive aneurysm management requires surgical teams skilled in both endovascular and open techniques. While EVAR is less invasive, it has not been shown to reduce long-term complications or mortality compared to open surgery [40]. In fact, endoleak rates after endovascular treatment of ACF can be as high as 50%, potentially leading to thrombosis or aneurysm sac expansion [41]. Endovascular repair also carries risks of device migration, branch artery occlusion, and IVC injury, so treatment decisions must be carefully tailored to each patient [42].

In our case, due to limited experience with endovascular treatment of ruptured common iliac artery aneurysms with ACFs and familiarity with open surgical techniques, we opted for open repair. The fistula was large, and the risk of postoperative endoleak was high. After collaborative planning between the anesthesiology and cardiac surgery teams, we decided to perform IABO under local anesthesia prior to general anesthesia, as the patient was hemodynamically unstable due to high-output heart failure.

In cases of ruptured aneurysms with ACFs, early diagnosis and interdisciplinary communication between anesthesiologists and cardiovascular surgeons are essential for effective treatment planning and anesthesia management. In this case, the patient avoided organ failure related to heart failure and recovered fully, with no neurological sequelae and a return to normal daily function.

## Conclusions

We presented a case of a ruptured aortic aneurysm with a fistula between the right common iliac artery and the IVC. Fistula plugging using IABO was performed prior to general anesthesia, rapidly reducing shunt blood flow and enabling early stabilization of high-output heart failure. In such cases, anesthetic management should aim to avoid excessive preload and sudden increases in afterload while maintaining adequate perfusion pressure until the fistula is controlled with IABO, following principles similar to the management of congestive heart failure.
